# Impact of the Mitochondrial Genetic Background in Complex III Deficiency

**DOI:** 10.1371/journal.pone.0012801

**Published:** 2010-09-17

**Authors:** Mari Carmen Gil Borlado, David Moreno Lastres, Maritza Gonzalez Hoyuela, Maria Moran, Alberto Blazquez, Rosa Pello, Lorena Marin Buera, Toni Gabaldon, Juan Jose Garcia Peñas, Miguel A. Martín, Joaquin Arenas, Cristina Ugalde

**Affiliations:** 1 Centro de Investigación, Hospital Universitario 12 de Octubre, Madrid, Spain; 2 Centro de Investigación Biomédica en Red de Enfermedades Raras-CIBERER, U723, Madrid, Spain; 3 Centre for Genomic Regulation-CRG, Barcelona, Spain; 4 Servicio de Neurología, Hospital Universitario Niño Jesús, Madrid, Spain; Universidad Europea de Madrid, Spain

## Abstract

**Background:**

In recent years clinical evidence has emphasized the importance of the mtDNA genetic background that hosts a primary pathogenic mutation in the clinical expression of mitochondrial disorders, but little experimental confirmation has been provided. We have analyzed the pathogenic role of a novel homoplasmic mutation (m.15533 A>G) in the cytochrome *b* (*MT-CYB*) gene in a patient presenting with lactic acidosis, seizures, mild mental delay, and behaviour abnormalities.

**Methodology:**

Spectrophotometric analyses of the respiratory chain enzyme activities were performed in different tissues, the whole muscle mitochondrial DNA of the patient was sequenced, and the novel mutation was confirmed by PCR-RFLP. Transmitochondrial cybrids were constructed to confirm the pathogenicity of the mutation, and assembly/stability studies were carried out in fibroblasts and cybrids by means of mitochondrial translation inhibition in combination with blue native gel electrophoresis.

**Principal Findings:**

Biochemical analyses revealed a decrease in respiratory chain complex III activity in patient's skeletal muscle, and a combined enzyme defect of complexes III and IV in fibroblasts. Mutant transmitochondrial cybrids restored normal enzyme activities and steady-state protein levels, the mutation was mildly conserved along evolution, and the proband's mother and maternal aunt, both clinically unaffected, also harboured the homoplasmic mutation. These data suggested a nuclear genetic origin of the disease. However, by forcing the *de novo* functioning of the OXPHOS system, a severe delay in the biogenesis of the respiratory chain complexes was observed in the mutants, which demonstrated a direct functional effect of the mitochondrial genetic background.

**Conclusions:**

Our results point to possible pitfalls in the detection of pathogenic mitochondrial mutations, and highlight the role of the genetic mtDNA background in the development of mitochondrial disorders.

## Introduction

Mitochondrial complex III (CIII, ubiquinol-cytochrome c reductase or cytochrome bc1 complex, E.C.1.10.2.2) is a multiprotein enzyme complex that catalyzes the transfer of electrons from reduced coenzyme Q to cytochrome c, with a concomitant translocation of protons across the inner mitochondrial membrane [1]. The purified bovine complex is a symmetric homodimer with a combined molecular mass of ∼450 kDa [2], [3]. Each monomer is composed of 11 subunits, of which ten are encoded in the nucleus and one (cytochrome b) in the mitochondrial genome. Respiratory chain complex III deficiency [MIM 124000] is a relatively rare cause of mitochondrial dysfunction. Mitochondrial DNA (mtDNA) mutations in the cytochrome *b (MT-CYB)* gene constitute a major cause of complex III deficiency, and underlie a wide range of neuromuscular disorders [4]. These include mitochondrial encephalomyopathy [5], hypertrophic or histiocytoid cardiomyopathy [6], [7], or sporadic mitochondrial myopathy, where exercise intolerance is the predominant symptom [8]. Additional features include blood acidosis, muscle weakness or myoglobinuria. Defects in *MT-CYB* are also documented in patients with multisystem disorders in cases of exercise intolerance accompanied by deafness, mental retardation, retinitis pigmentosa, cataract, growth retardation, and epilepsy [9], [10]. Mutations in *MT-CYB* have also been associated with Leber hereditary optic neuropathy (LHON) [MIM: 535000], a maternally inherited disease resulting in acute or subacute loss of central vision due to optic nerve degeneration [11].

When a new mutation is detected in the mtDNA of a patient with a mitochondrial defect, a strict association between the mutation and the cellular dysfunction must be established before assuming pathogenicity. Although many mutations in mitochondrial tRNA genes and coding regions have been shown to cause diseases, the high rate of evolution of mtDNA creates many new polymorphic sites. For this reason, a number of useful criteria have been proposed in order to avoid the mistake of incorrect assignment of pathogenicity to a mutation [12]. These criteria generally include the following aspects: i) the mutation should be preferably found in heteroplasmic state; ii) there should be a strong association between heteroplasmic levels, clinical symptoms, biochemical data, and family history; iii) the mutation should be highly conserved among species; and iv) transmitochondrial cybrids should not restore the cellular and biochemical defects in mitochondrial function found in the original tissue; when a defective mitochondrial function is maintained in cybrid cells bearing mutated mtDNA, it is generally assumed that the mutation is likely to be a pathogenic cause of disease. Additionally, in recent years a number of studies emphasize the importance of the mtDNA genetic background that hosts a primary pathogenic mutation in the clinical expression of mitochondrial disorders. The clearest example is the preferential association of the Eurasian haplogroup J with the 11778/*ND4* and 14484/*ND6* LHON pathogenic mutations [13].

In this work we report a complex III-deficient patient with early-onset metabolic acidosis and seizures, who harboured a novel m.15533 A>G homoplasmic mutation in the *MT-CYB* gene. Although this genetic variant did not easily fulfil the pathogenicity criteria for mtDNA mutations, by forcing the *de novo* functioning of the OXPHOS system in mutant transmitochondrial cybrids we have demonstrated a direct functional effect of the mtDNA genetic background on the biogenesis of the mitochondrial respiratory chain.

## Materials and Methods

### Ethics Statement

This study was approved by the institutional ethics committee (Hospital Universitario 12 de Octubre, Madrid, Spain), and was in accordance with the Declaration of Helsinki for Human Research. The patient's mother has given written informed consent (as outlined in the PLoS consent form) to publication of the case details.

### Case Report

The proband was a male born to non-consanguineous parents, delivered at full term by caesarean after an uneventful pregnancy. At age 23 days he presented with abnormal movements of his arms, and generalized hypertonia. He also showed central cyanosis with episodes of apnea that required intubation and mechanical ventilation for twelve days, and a convulsive status with tonic-clonic seizures, which were recurrent during the following two days. After treatment with antiepileptic drugs, the paroxysmal movements finally remitted. Brain computerized tomography scans and brain ultrasonography revealed no abnormalities. Laboratory examinations revealed persistent metabolic acidosis that was treated with bicarbonate, elevated anion gap, and moderate increase of resting plasma lactate (3.4 mmol/L; normal <2). At age two years a muscle biopsy was taken, which showed a single mitochondrial respiratory chain complex III defect. Other studies discarded pyruvate decarboxylase deficiency and organic acidemia. At three years of age brain magnetic resonance imaging (MRI) displayed small abnormal signals in both thalami, and diffuse hypersignals in T2-weighted sequence in the white matter of the peripheral area of atrium as a consequence of myelination delay. At the age of six he presented with developmental delay affecting his speech skills, which started to be treated by language therapy. He also developed behaviour disturbances and a functional clubfoot. Brain MRI and MR spectroscopy were normal. At present, at age nine years, he has a mild mental and developmental delay, functional clubfoot, cognitive abnormalities, attention difficulties and hyperactivity, initiating treatment with methylphenidate. His parents and a maternal aunt were all asymptomatic. His mother suffered a previous miscarriage at two months of gestation. A maternal aunt was referred to die at two months of age from an unknown disease.

### Cell Cultures

Fibroblasts were obtained from skin biopsies and cultured in DMEM medium (Life Technologies) supplemented with 10% foetal calf serum (FCS) and 100 IU/ml penicillin and 100 IU/ml streptomycin. Transmitochondrial cybrids were obtained by fusion of 143B206 TK^-^ rho zero cells with control or patient-derived enucleated fibroblasts or platelets as previously described [14]. To block mitochondrial translation, 15 µg/ml doxycycline were added to the culture medium. Cells were grown in exponential conditions and harvested at the indicated time points.

### Enzyme activities of mitochondrial respiratory chain complexes

Mitochondrial respiratory chain enzyme activities were measured in muscle (quadriceps muscle biopsy), skin fibroblasts, and cybrids as described before [15], and expressed relative to the citrate synthase activity in muscle, and as their specific activities in fibroblasts and cybrids (Table 1).

**Table 1:** Residual enzyme activities of mitochondrial respiratory chain complexes in different tissues from the index patient.

	Muscle*		Fibroblasts**		Cybrids**	
	*Patient*	*Control Range (n = 100)*	*Patient*	*Control Range (n = 9)*	*Mutants Mean (n = 6)*	*Control Range (n = 5)*
CI	17,1	10–30	35,1	26–50	10	4–17
CII	16,3	4,5–17,4	10	10–17	12	11–27
CI+CIII	14,5	8.5–26	**122**	223–750	37	32–96
CII+CIII	nd	-	**2,7**	4,4–18,5	2,8	2,6–7,3
CIII	**14,6**	28–98	**15,1**	29–87	**74**	13–71
CIV	22,7	16–80	**15,2**	49–128	**39**	20–36
CS	194	78–250	61	60–160	142	108–175

Enzyme activities are expressed as

*cU/U citrate synthase (CS) and

**nmol.min^−1^.mg prot^−1^. CS activity is expressed as mU/mg protein. Abnormal values are indicated in bold. nd, not determined. Complex I, CI; Complex II, CII; Complex III, CIII; Complex IV, CIV.

### Genetic analyses

Total DNA was extracted from blood, muscle, fibroblasts and cybrids by standard methods. The whole mitochondrial DNA (mtDNA) from patient's muscle was sequenced by SECUGEN S.L. (Madrid, Spain). The complete mtDNA was amplified from total DNA in 24 overlapping 800–1,000 bp-long polymerase chain reaction (PCR) fragments. The PCR fragments were sequenced in both strands in an ABI 3730 sequencer using a BigDye v3.1 sequencing kit (Applied Biosystems, Foster City, CA), and were compared with the revised Cambridge reference sequence, CRS [16] (Table 2). Discrepancies were analyzed in the human mitochondrial genome databases MITOMAP (www.mitomap.org), mtDB (www.genpat.uu.se/mtDB/), and GiiB-JST mtSNP (http://mtsnp.tmig.or.jp/mtsnp/index_e.shtml). To quantify the heteroplasmy levels of the m.15533 A>G mutation, PCR-restriction fragment length polymorphism (RFLP) analysis was performed in the proband's family using the following primers: 5′-CACTATTCTCACCAGACCTC-3′, (forward) and 5′-ACGCCTCCTAGTTTGTTAGG-3′ (reverse), and digestion with the restriction enzyme *Dde*I (New England Biolabs). Two hundred healthy control subjects of Spanish background obtained from the Blood Bank of our Institution were screened to rule out the presence of mutations in the healthy population.

**Table 2:** Haplogroup affiliation and non-synonymous nucleotide changes of the mtDNA sequence from the index patient.

Genbank ID	HAPLOGROUP	Non-synonymous polymorphisms relative to rCRS^a^	Amino acid Change
HM046248	H2	A8860G (*ATP6*)	Thr>Ala
		G9477A (*CO3*)	Val>Ile
		C14766T (*CYB*)	Thr>Ile
		A15326G (*CYB*)	Thr>Ala
		A15533G (*CYB*)	Asn>Asp

a rCRS refers to the revised Cambridge reference sequence [16], which belongs to haplogroup H2a.

Additionally, sequencing ruled out mutations in the coding regions of the following complex III nuclear genes: *CYC1*, *UQCRFS1*, *UQCRQ*, *UQCRH*, *UCRC*, and *BCS1L*.

### Bioinformatic analyses

Cytochrome b protein sequences from diverse vertebrate species were downloaded from Uniprot (The Uniprot Consortium, 2010)[17] [17], and aligned with MUSCLE v3.2 [18]. The potential effect of the N263D and N260D mutations was evaluated by homology modelling of the mutated sequences using the 3D structure of bovine complex III stored in PDB (1NTM) as a template. Modelling was performed at the Swiss model server [19]. 3D models were visualized using Chimera [20].

### Quantification of the mtDNA copy number

Relative quantification of mtDNA versus nuclear DNA (nDNA) was performed by real-time PCR in a HT 7500 Real Time PCR System (Applied Biosystems, Foster City, CA, USA) as previously described [21], except that the forward primer used for mtDNA amplification was: 5′-CCA CGG GAA ACA GCA GTG AT-3′. Results were presented as mean ±SD values.

### Mitochondrial Protein Isolation and Blue Native Analysis

Blue native methods were performed as previously described [21]. Primary antibodies (Molecular Probes) were raised against the complex I NDUFS3 and NDUFA9 subunits, complex III core2 protein, complex IV COX2 and COX5A subunits, and complex II SDHA subunit. Peroxidase-conjugated anti-mouse IgG was used as secondary antibody (Molecular Probes). The signal was detected with ECL® plus (Amersham Biosciences).

## Results

### Genetic and biochemical analyses of the *MT-CYB* m.15533A>G mutation

**Figure 1:**
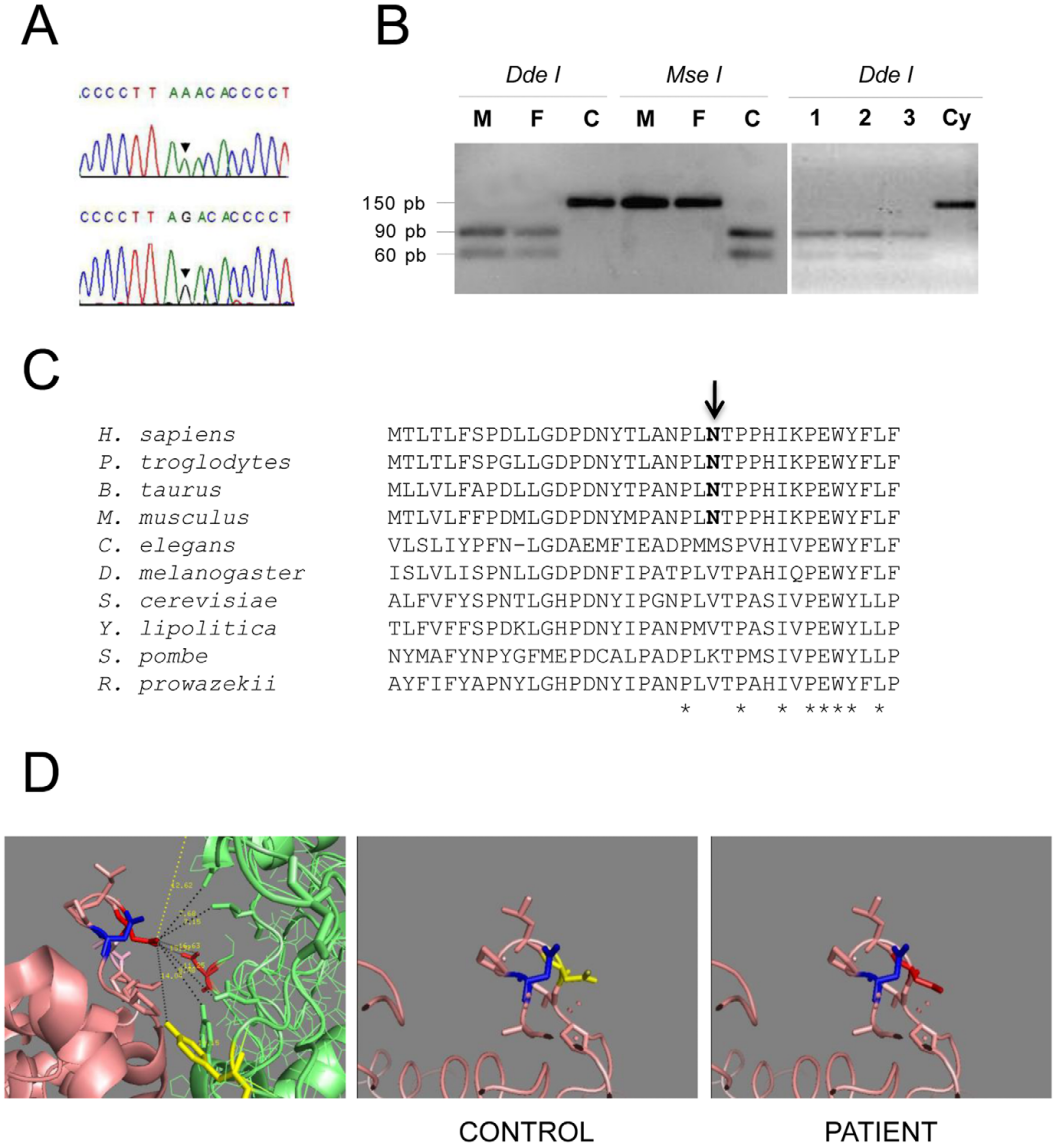
Genetic and structural analysis of the m.15533A>G mutation. (A) Electropherogram showing the nucleotide change in patient's muscle DNA, indicated by arrowheads. (B) PCR-RFLP analysis of the *MT-CYB* mutation. Uncut (wild-type) DNA consists of a 150 bp PCR product. The mutated sequence contains one *Dde*I restriction site that results in two products of 90 and 60 bp after digestion. The control sequence contains one *Mse*I restriction site that results in the same two products after digestion. C, wild-type control; F, proband's fibroblasts DNA; M, proband's muscle DNA; Cy, control cybrid; 1, 2 and 3 refer to three independent mutant cybrid clones. (C) Alignment of cytochrome b amino acid sequences from selected species. Asparagine at amino acid position 263 is indicated with an arrow. (D) Partial 3D-images of the cytochrome b protein. The left panel shows the interaction site between the asparagine 263 (indicated in red) of cytochrome b and the aspartate 2 of the cytochrome c1 subunit (coloured in green). This is the closest negatively charged side-chain of cytochrome c1, which is facing the same aqueous pocket at a distance of 11.25 Armstrongs. Asparagine at position 260 of cytochrome b is indicated in blue. The central panel shows the 3D-structure of the asparagine 263 (in blue) in controls. The right panel shows the predicted structural effect of the N263D substitution (in red) in the patient. The images were obtained using the Chimera software [20].

Mitochondrial respiratory chain enzyme activities showed a single complex III defect in the patient's skeletal muscle (52% of the lowest control value), and a combined deficiency of complexes III and IV in fibroblasts (52% and 31% of the lowest control values respectively) (Table 1). Sequencing of the entire mitochondrial genome in patient's muscle enabled easy identification of haplogroup-specific polymorphisms and novel private substitutions (Table 2), and confirmed that the patient belonged to the European haplogroup H2 (GenBank sequence accession number HM046248). Comparison of the sequence with the revised Cambridge reference sequence (rCRS) revealed 12 synonymous substitutions, 5 non-synonymous substitutions, and 19 substitutions either in mitochondrial rRNAs, tRNAs or in the mtDNA D-loop region. One missense mutation in *MT-CYB* (m.15533A>G) was not previously reported in the mtDNA sequence databases, and it was not detected in 200 ethnically-matched controls (Figure 1A). The relative mutant load for this new nucleotide variant was measured by PCR-RFLP analysis in blood (not shown), muscle and fibroblasts of the patient (Figure 1B), and showed homoplasmic levels of the mutation in all tissues. *Dde*I-RFLP analysis also showed that the *MT-CYB* mutation was homoplasmic in blood from the proband's mother and maternal aunt, both clinically unaffected (not shown).

To discriminate between a nuclear or mitochondrial origin of the disease, transmitochondrial cybrids were constructed by transferring mitochondria from patient's fibroblasts into 143B206 TK^-^ rho zero (ρ°) cells, which lack mtDNA. Ten independent cybrid clones retained homoplasmic loads of the m.15533A>G mutation (Figure 1B). The enzyme activities of all respiratory chain complexes were normal in six independent mutant clones (Table 1), which would in principle indicate that the main genetic defect should be located in the nuclear genome.

### Features of the *MT-CYB* m.15533A>G mutation

The m.15533A>G transition changes an asparagine into aspartate at amino acid residue 263, within an amino acid stretch relatively conserved amongst mammals, but not further on in evolution (Figure 1C). Based on its crystal structure [2], [3], the cytochrome b subunit consists of eight intramembrane spanning domains (helixes A–H). Asn 263 is located between the E–F transmembrane helixes, within the second half of loop ef of cytochrome b, in close contact with functional domains of the RISP and cytochrome c1 subunits. The N263D amino acid substitution would exchange a positive for a negative charge within this region, possibly affecting the interaction between cytochrome b and the RISP and cytochrome c1 subunits (Figure 1D). These findings suggested that the N263D mutation could have an effect on the structural integrity of mitochondrial respiratory chain complex III. However, a structural analysis of the N263D substitution only predicted a mild effect on the interaction between complex III subunits. As shown in Figure 1D, residue 263 (shown as red or yellow residues on the pink-coloured chain) is facing an aqueous pocket, and the distances to the closest residues of cytochrome c1 (coloured in green) are larger than 7 Armstrongs. Several other charged residues, an arginine and three aspartates, are present at close distances, suggesting that the relative charge introduced by the mutation is not large. A similar asparagine to aspartate substitution at amino acid position 260 of cytochrome b (shown in blue in the figure) was previously reported as a polymorphism [22]. The side chain of this residue is facing the same aqueous pocket in a very close position to that of 263. Altogether, these data do not plead in favour to consider the N263D as a true pathogenic mutation.

### Normal steady-state levels of respiratory chain complexes in MT-CYB mutant cells

**Figure 2:**
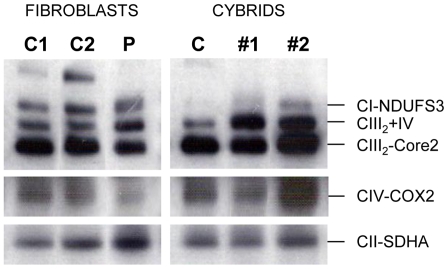
BN-PAGE analysis of mitochondrial respiratory chain complexes in control and mutant fibroblasts and cybrids. Mitochondrial particles were isolated as described in Methods and 40 µg of protein were analyzed on a 5–15% BN-polyacrylamide gel for the separation of multisubunit complexes. Western-blot analysis was performed using antibodies against the indicated OXPHOS subunits. CI, fully-assembled complex I. CIII_2_, complex III dimer. CIV, complex IV. CIII_2_+IV indicates the presence of the supercomplex containing complexes III and IV. C1 and C2, control fibroblasts. P, patient's fibroblasts. C, control cybrid. Two independent mutant cybrids are indicated as #1 and #2.

To analyze the respiratory chain content in digitonin-extracted mitochondria from patient's fibroblasts and cybrids, 1D-BN-PAGE analysis was combined with complex I *in-gel* activity assay and western blot with antibodies raised against specific OXPHOS subunits (Figure 2). In patient's fibroblasts low steady-state levels of respiratory chain complexes III and IV were observed, suggesting an impaired assembly/stability defect. Consequently, decreased enzyme activities of these complexes were found in this tissue (Table 1). As expected from their restored enzyme activities, the mutant cybrids displayed normal steady-state levels of respiratory chain complexes relative to the controls.

### Delayed assembly kinetics of respiratory chain complexes in mutant cybrids

**Figure 3:**
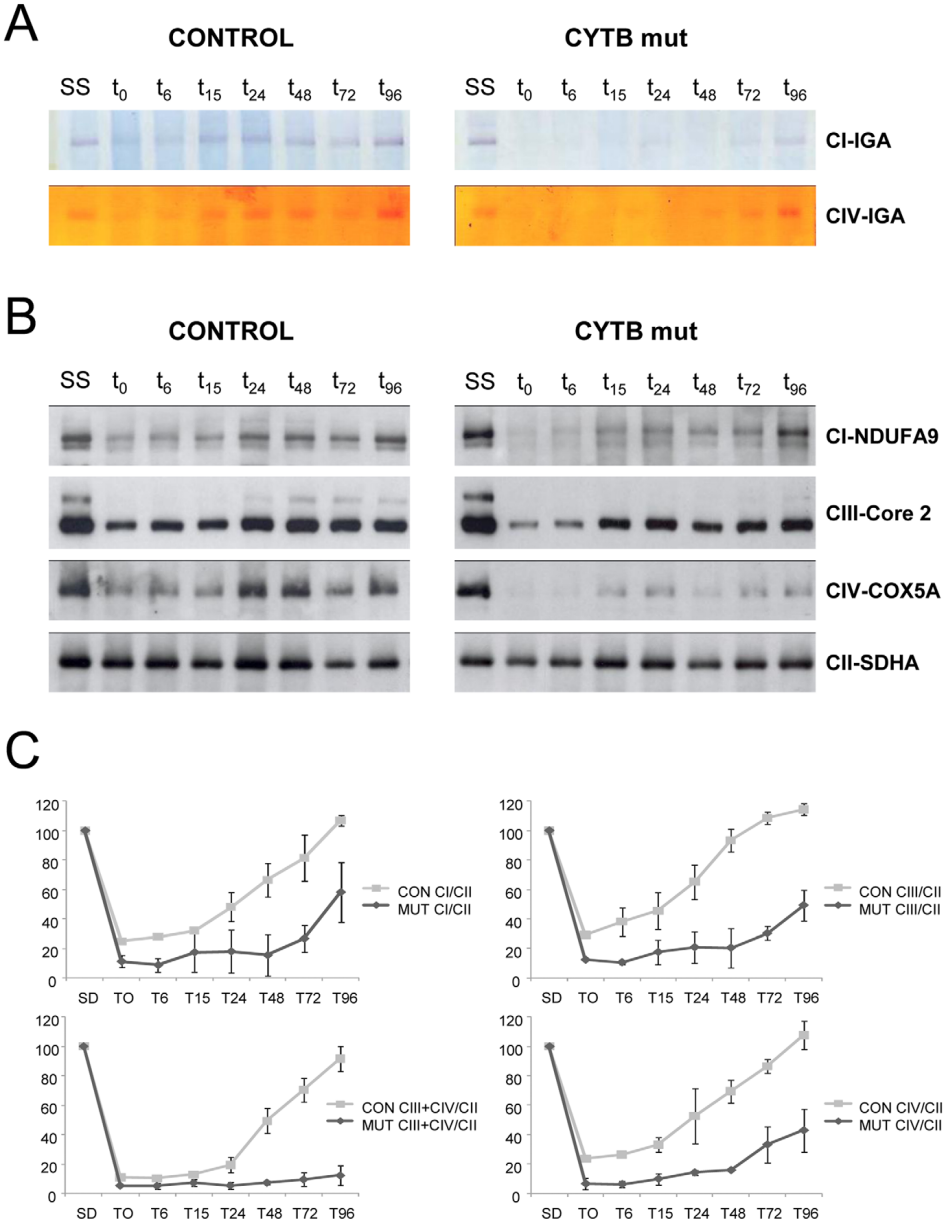
Assembly kinetics of respiratory chain complexes in control and mutant cybrids. Two different control cybrids belonging to haplogroup H, and two independent *MT-CYB* mutant clones were treated for 6 days with doxycycline (an inhibitor of mitochondrial translation), the medium was replaced by doxycycline-free medium and cells were collected at 0, 6, 15, 24, 48, 72, and 96 hours (indicated as t0–t16). SS indicates the steady-state expression levels of the respiratory chain complexes (A) Example of one control and one mutant clone. 40 µg of crude mitochondrial pellets were analyzed by BN-PAGE in combination with complex I and complex IV-IGA assays. (B) Duplicate gels were blotted and incubated with antibodies against the NDUFA9 complex I subunit, complex III core2 protein, complex IV COX5A subunit and complex II SDHA subunit. (C) The signals from the blots were quantified, expressed as percentage of the untreated cells (SS), normalized with the complex II SDHA subunit and plotted. The restoration curves constitute the mean values ± SD obtained from the two controls and the two independent mutant cybrids. Upper left panel, complex I assembly rates. Upper right panel, complex III assembly rates. Lower left panel, complex IV assembly rates. Lower right panel, supercomplex CIII_2_+IV assembly kinetics.

Next, we reversibly blocked for 6 days mitochondrial protein translation with doxycycline, thus depleting the cells of OXPHOS complexes that contained mitochondrial-encoded subunits [23]. After the release of drug inhibition, mitochondrial translation resumed and the assembly of newly-synthesized mitochondrial complexes was investigated by collecting cells at different time points (0, 6, 15, 24, 48, 72, and 96 hours). Digitonin-isolated mitochondria were analyzed by BN-PAGE electrophoresis in combination with *in-gel* activity assays (Figure 3A) or Western-blot using antibodies against subunits from different OXPHOS complexes (Figure 3B). The signals obtained from the Western-blots were quantified, normalized for the expression levels of mitochondrial complex II, expressed as a percentage of the untreated cells (which correspond to the steady-state expression levels), and calculated the average numerical values for the restoration curves of the respiratory complexes in control and mutant cybrids (Figure 3C). A significant delay in the recovery kinetics of respiratory chain complexes I, III and IV was observed in the two independent mutant cybrids relative to two controls of the same haplogroup (Figure 3C).

The mtDNA copy number was calculated for control and mutant cybrids in order to exclude the possibility that the differences observed in the assembly kinetics of respiratory chain complexes could be due to variations in the mtDNA content. No significant differences were found in the mtDNA/nDNA ratio between the controls (292±35) and the mutants (289±38) belonging to haplogroup H. Taken as a whole, our results demonstrate a functional effect of the mtDNA genetic background of the mutant clones in the assembly kinetics of respiratory chain complexes.

## Discussion

In this work we have shown the pathogenic role of the novel m.15533A>G genetic variant in the *MT-CYB* gene in a patient with metabolic acidosis and hyperlactacidemia, seizures, mild mental delay, behaviour disturbances and a single enzyme defect of mitochondrial respiratory chain complex III. Based on the current standard pathogenicity criteria for mtDNA mutations, the mutation would have been easily excluded as potentially deleterious. Our patient harboured the mutation in homoplasmic state, as did his clinically unaffected mother and maternal aunt. The mutation was not highly conserved along evolution and the predicted structural data did not plead in favour of a deleterious effect of the N263D substitution on the structure of cytochrome b. Although he showed defective respiratory chain enzyme activities and protein levels in muscle and cultured fibroblasts grown in exponential conditions, these respiratory chain enzyme activities and assembly defects were restored to normal in transmitochondrial cybrids harbouring the homoplasmic m.15533A>G mutation, when these cells were grown in a favourable glucose-containing medium under no stress conditions. Only when the mutant cybrids were forced to the *de novo* building up and functioning of the OXPHOS system by treating the cells with doxycycline, we were able to observe a drastic functional effect of the mtDNA genetic background on the assembly kinetics of the respiratory chain complexes.

Similar observations were previously made by our group in cybrid cell lines bearing the three classical homoplasmic LHON mutations [21]. These cells neither showed respiratory chain enzyme defects nor decreased steady-state levels of OXPHOS complexes, but displayed differentially-delayed assembly rates of respiratory chain complexes I, III, and IV amongst mutants belonging to different mtDNA haplogroups. Likewise, the mtDNA genetic background was recently shown to play an important role in modulating the bioenergetics and biochemical defects in cybrids bearing the *ATP6* NARP/MILS mutation [24]. This is in agreement with the proposed effect on protein function of single nucleotide polymorphisms (SNPs) that are located in the coding region, which could affect protein stability, ligand binding, catalysis, allosteric regulation and post-translational modifications [25]. Basal differences in OXPHOS capacities such as mitochondrial transcription and replication, protein synthesis, and bioenergetics have recently been demonstrated in control cybrids from different mtDNA haplogroups [26], [27]. These results reveal that specific mtDNA polymorphisms may modify the pathogenic potential of mtDNA mutations by affecting the overall biogenesis and function of the OXPHOS complexes.

In our *MT-CYB* mutant cybrids, the m.15533A>G mutation most likely hampers the proper assembly of respiratory chain complex III, which would further affect the formation of complex I [28]. In addition, the mutant clones showed, besides strong complex I and complex III assembly defects, a severe impairment of complex IV and supercomplex CIII_2_+CIV assembly rates. This effect could be explained by the presence of two mtDNA polymorphisms in the *MT-CO3* and *MT-ATP6* genes, G9477A and A8860G respectively, plus two additional mtDNA polymorphic variants in the *MT-CYB* gene, C14766T and A15326G, (Table 2) which could add a deleterious effect to that caused by the m.15533A>G mutation. Our results, however, do not explain the differences in the clinical expression of the m.15533A>G mutation among members of the proband's family. Although reduced penetrance is a relatively common feature of homoplasmic mtDNA mutations leading to mitochondrial disorders [29], [30], it suggests the requirement of additional contributors to the clinical manifestations of the disease. For instance, most patients with LHON disease carry homoplasmic mtDNA mutations. Although all offspring inherit the mutation, often only some family members will develop the disease [30], which emphasizes the importance of nuclear genetic, epigenetic or environmental factors as phenotypical modulators of mitochondrial disorders.

In general, the mutations that have the most deleterious effect on mitochondrial function will cause obvious clinical phenotypes. The m.3243A>G mutation, for example, usually causes a devastating multisystem disease and fulfils all the criteria for pathogenicity [31]. The problems may arise when a mutation causes disease only in the presence of an additional factor, or when it only causes a mild clinical phenotype as it occurs in our patient. In these cases the ‘conventional’ criteria might not be applicable, and deeper functional tests should be performed in cybrid cell lines that harbour the mutation of interest, including i.e. oxygen consumption and mitochondrial protein synthesis analyses, or *de novo* restoration of enzyme activities and assembly of the various components of the oxidative phosphorylation system. In this sense our study describes a useful mechanism to unveil potentially deleterious mutations in patients with signs of mitochondrial disease but apparently normal respiratory chain enzyme activities. Besides, our results point to possible pitfalls in the detection of pathogenic mitochondrial mutations, and highlight the potentially important effects of the genetic mtDNA background in the development of mitochondrial disorders, which should be taken into account when defining the pathogenicity criteria for mtDNA mutations.
